# Establishing a UHPLC-MS/MS method for evaluation of the influence of stir-frying on the pharmacokinetics of seven compounds in Arctii Fructus

**DOI:** 10.1039/d2ra03637a

**Published:** 2022-09-27

**Authors:** Yun Shi, Jing Hu, Hongsen Wang, Zhankuan Yan, Guangrui Zhao, Xun Gao, Weidong Li, Kunming Qin

**Affiliations:** School of Pharmacy, Nanjing University of Chinese Medicine Nanjing 210046 PR China liweidong0801@163.com; School of Pharmacy, Jiangsu Ocean University Lianyungang 222005 PR China qinkm123@126.com; Department of Pharmacy, People's Hospital of Macheng City Macheng 438300 PR China; Jiangsu Yuanchuang Pharmaceutical Research and Development Co., Ltd. Lianyungang 222005 PR China

## Abstract

At a fundamental level, the broad application of raw and stir-fried Arctii Fructus products as anti-tumor and anti-inflammatory agents is commonly recognized. In order to understand some of the discrepancies pertaining to their therapeutic functions, an associated study of pharmacokinetics is required. In this study, a reliable ultra-high performance liquid chromatography-tandem mass spectrometry (UHPLC-MS/MS) method was initially developed for the concurrent determination of seven compounds from Arctii Fructus in plasma. By virtue of its acceptable performance, the developed method was incorporated in assessing the pharmacokinetic differences of the compounds following the oral administration of raw and stir-fried Arctii Fructus. Subsequently, the results highlighted potential improvements to the exposure of the seven compounds, and the enriched bioavailability of arctiin through the process of stir-frying, which are deemed essential constituents of Arctii Fructus. This study represents the initial attempt at assessing the influence of stir-frying on the pharmacokinetic behaviors of the primary Arctii Fructus composition. Furthermore, the results could be instrumental in expanding the clinical applications of diverse Arctii Fructus products, and reveal the inherent processing mechanism.

## Abstract 2

The pharmacokinetics of multiple, orally administrated compounds of raw and stir-fried Arctii Fructus extracts using a UHPLC-MSMS method.

## Graphical abstract

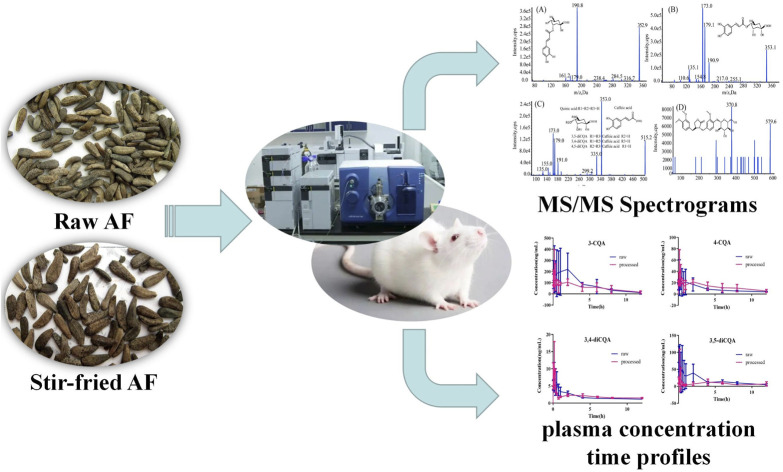

## 1. Introduction

Historically, the therapeutic value of Arctii Fructus (AF, known in China as Niubangzi) and the dried fruits of *Arctium lappa* L. are commonly used as a remedy for ailments such as colds and influenza in traditional Chinese medicines because of their antiviral and anti-inflammatory properties.^1^ In general, the key components of AF include the main lignans such as arctigenin (ARG), arctiin (ARC), lappaol and matairesinol.^2^ Additionally, supplementary phytochemicals such as caffeic acid, chlorogenic acid (3-CQA), and isochlorogenic acids were also reported.^3^ Among the previously mentioned active components, ARG and its glycoside ARC have been reported to exhibit antioxidant, anti-tumor, and anti-inflammatory characteristics which are critical aspects of therapeutic agents.^4,5^ Furthermore, ARG (a representative of the dibenzylbutyrolactone lignans) has been reported to encompass diverse bioactivities and critical pharmacological properties.^6^

Principally, the intricate processing of traditional Chinese medicine (TCM) functions as a pharmaceutical method for treatment purposes. According to the theory of TCM, medicinal substances require special treatment methods such as stir-frying, steaming, calcining, or boiling in order to enhance their curative effects and reduce toxicity.^7^ According to the Chinese Pharmacopoeia, there exist two forms of AF: raw Arctii Fructus (RAF) and stir-fried Arctii Fructus (SAF).^8^ Upon processing the medicinal agents, changes in their chemical compounds in the form of increase or reduction of content, and the formation or disappearance of specific compounds were observed.^9,10^ Notably, the preceding reports highlight an increase in ARG content and an overall decrease in ARC after the stir-frying process.^11^ Nevertheless, the functioning mechanism of the activity changes remained obscure even after the process of stir-frying *in vivo*.

In the past few years, important chemical studies on the topic of AF have emphasized the qualitative analysis of its major components using various analytical methods such as HPLC, LC-MS/MS, and so on.^12,13^ Specifically, the study of pharmacokinetics pertaining to the differences of the main compounds between RAF and SAF awaits further reports. In general, pharmacokinetics is deemed an effective method of ascertaining the influence of processing on the components absorbed into the body following oral administration in rats.^14,15^ Consequently, the evaluation of major compounds by pharmacokinetics to elucidate the mechanism of activity change by comparing the pharmacokinetic properties of the processing approaches for AF is acknowledged to be essential.

In the current research, a systematic and validated method was formulated for the simultaneous quantification of seven compounds, 3-CQA, cryptochlorogenic acid (4-CQA), isochlorogenic acid A (3,5-diCQA), isochlorogenic acid B (3,4-diCQA), isochlorogenic acid C (4,5-diCQA), ARC, and ARG using pharmacokinetics. Additionally, the study of multiple compounds in rat plasma was assessed in a pharmacokinetic study of RAF and SAF. Furthermore, the proposed method was effectively used to determine the necessary pharmacokinetic outlines for the clinic research of stir-fried AF.

## 2. Experimental

### 2.1. Chemicals and reagents

In particular, reference substances of 3-CQA, 4-CQA, 3,5-diCQA, 3,4-diCQA, 4,5-diCQA, ARC, ARG, and tinidazole (internal standard, IS), all with a purity value of over 98.0%, were obtained from Chengdu Pusi Biological Company (Chengdu, China). In addition, the acetonitrile (LC/MS grade) and formic acid (UHPLC grade) were procured from Dikma Technologies (Foothill Ranch, CA, USA). Ultra-pure water was obtained using a Milli-Q system (Millipore, Boston, MA, USA), and the Arctii Fructus was purchased from the Bozhou Medicinal Herb Company (Anhui province, China). All the other reagents were of analytical grade.

### 2.2. Apparatus and UHPLC-MS/MS conditions

All the components were measured using a Shimadzu UHPLC system (Shimadzu, Japan), and a Triple Quadrupole 5500 LC-MS/MS system (AB Sciex, USA) in negative ion mode. Notably, acetonitrile–methanol (4 : 1) (B) and 0.1% formic acid–water (A) were utilized to separate the analytes on an Agilent Zorbax Eclipse Plus C18 column (2.1 mm × 100 mm, 1.8 μm) with a flow rate of 0.3 mL min^−1^. The gradient program was: 5–28% B (0–3 min), 28% B (3–3.5 min), 28–38% B (3.5–4 min), 38% B (4–4.5 min), 38–100% B (4.5–6 min), 100–5% B (6–7 min). With a column temperature of 40 °C, the optimized key parameters including curtain gas, collision gas, ion spray voltage, and temperature were assigned values of 55 psi, 35 psi, 4500 V, and 550 °C, respectively.

### 2.3. Preparation of the RAF and SAF extracts

In accordance with the 0213 guidelines stipulated in the Chinese Pharmacopeia (2020 edition), the stir-frying protocols for AF were followed exactly during the entire process. Initially, the RAF was heated with constant stirring until it was observed that the AF had a yellow appearance with a slight crackle and a light fragrance. Then, the SAF was discharged onto a plate to cool before the following experiment.

Subsequently, both RAF (0.12 kg) and SAF (0.12 kg) were accurately weighed, and in tandem an extraction solvent was added and independent refluxing of all three components (70% ethanol) followed with a material : solvent ratio of 1 : 8 for 1 h during each iteration. Under reduced pressure, the extracted solutions were concentrated to 100 mL using a rotary evaporator and were stored at 4 °C for future use.

### 2.4. Preparation of standard and quality control (QC) samples

Initially, stock solutions (1 mg mL^−1^) were prepared by dissolving individual standard substances in methanol. Afterwards, they were serially diluted to derive working solutions for the calibration curves and QC samples. Tinidazole was selected as the internal standard (IS) because of its good acidity (p*K*_a_ = 2.30 ± 0.34), solubility and good mass spectrum response. Finally, the IS solution was prepared in methanol at a concentration of 100 ng mL^−1^. All the solutions were stored at 4 °C.

### 2.5. Preparation of the plasma samples

The rat plasma sample (100 μL), IS (10 μL), and 2 M HCl (20 μL) were mixed and vortex mixed for 3 min. Subsequently, 1 mL of ethyl acetate–butyl alcohol (1 : 1) was spiked into the mixed solution, and then centrifuged for 5 min at 25 000 × *g*.^14^ Next, the supernatant was transferred and evaporated with nitrogen gas. These residues were then re-dissolved in 100 μL of 60% methanol, with the final solutions being vortex mixed for 3 min, and then centrifuged for 10 min at 25 000 × *g*. Finally, the supernatant was injected into the UHPLC-MS/MS for extensive analysis.

### 2.6. Method validation

#### 2.6.1. Linearity and lower limit of quantification (LLOQ)

The calibration curve was derived by adding standard solutions to the blank rat plasma. Statistically, the concentration ranges of the standard solutions were 2–1250, 2–2000, 1–2000, 1–500, 1–500, 2–10000, and 2–2000 ng mL^−1^ for 3-CQA, 4-CQA, 3,5-diCQA, 3,4-diCQA, 4,5-diCQA, ARC, and ARG, respectively. The linearity was evaluated using the peak area ratio of the seven compounds to their concentration in plasma with weighting factor of 1/*X*^2^ by least-squares linear regression. The LLOQ was defined as the lowest drug concentration at which both precision and accuracy were less than or equal to 20%.

#### 2.6.2. Selectivity

Chromatograms of blank plasma, blank plasma spiked with seven standards and IS, as well as rat plasma samples obtained after oral administration of RAF and SAF extracts were compared to evaluate the selectivity of the method. All samples were observed to have no endogenous substance interferences at the retention time of the analyte and the IS. The results showed that this method had good selectivity.

#### 2.6.3. Accuracy and precision

Over the course of three days, the QC samples from six different batches at three varying levels (low, medium, and high) were incorporated to gauge the intra-day accuracy and effectiveness. Evidently, the assessed index named RSD, in tandem with the percentage ratios of the calculated concentration to the nominal concentration. The intra- and inter-day precisions were expressed as RSDs, which should not exceed 15%.

#### 2.6.4. Stability

The overall stability (including freeze–thaw cycles, auto-sampling, and long-term stability) of all the analytes were assessed by measuring QC samples at three different concentrations. In particular, all the solutions were maintained at a constant 4 °C.

#### 2.6.5. Recovery and matrix effect

The extraction recovery was determined at three different QC levels using six replicates, by comparing the peak areas from the extracted QC samples with those obtained from the pure reference standards spiked in post-extracted blank rat plasma at the same concentrations. The matrix effect was evaluated by comparing the peak areas of the analytes obtained from the plasma samples with the analytes spiked after extraction, to those from the neat standard solutions at the same concentration. The acceptance criterion for the precision of recovery and matrix effect was ±15% RSD at all concentration levels.

### 2.7. Pharmacokinetic and data analysis

Firstly, male Sprague-Dawley rats (200–220 g) in the animal laboratory at the Nanjing University of Chinese Medicine under the standard conditions (License number: SYXK(SU)-2007-0030) were fed. The rats were fed and provided with water for one week prior to the experimentation. Secondly, before the administration of the extracts, the rats were stopped from eating but were provided with water for 12 h prior to the test. It was notable that the extraction yield of the medicinal substances was directly influenced by the process. In view of assessing the processing effects on the metabolic process of active ingredients *in vivo*, an identical amount of RAF was treated and administered to the rats. Depending on the clinical dosage administered to the human body, the dosage of RAF and SAF comprised 12 g kg^−1^ for oral administration. In addition, 500 μL blood samples (about 250 μL plasma) were collected in 1.5 mL heparinized polythene tubes at 0, 0.033, 0.083, 0.167, 0.33, 0.5, 0.75, 1, 2, 4, 6, 8, 12, 24, and 48 h *via* the postorbital venous plexus veins, after oral administration of the two extracts was observed. Finally, the collected plasma was centrifuged for 5 min at 25 000 × *g* and stored at −20 °C before the analysis. All the pharmacokinetic parameters were evaluated with the DAS 1.0 system (Medical College of Wannan, China). Furthermore, the maximum concentration of oral administration of AF (*C*_max_) and the time to reach *C*_max_ (*T*_max_) were derived from the concentration–time profile. All the values were given as mean ± standard deviation.

## 3. Results and discussion

### 3.1. Internal standard (IS) selection

It is necessary to use an IS to obtain high accuracy and precision when a mass spectrometer is equipped with LC as a detector, as it compensates for unavoidable assay variance in extraction efficiency, ionization effects, and transfer losses. The selected internal standard, tinidazole, had a similar retention time compared to the 4,5-diCQA and exhibited an optimum response and acceptable peak shape. Furthermore, tinidazole was not a metabolite of 4,5-diCQA *in vivo*. Thus, in this work it was appropriate to use it as an IS.

### 3.2. Optimization of UHPLC-MS/MS conditions

**Fig. 1:**
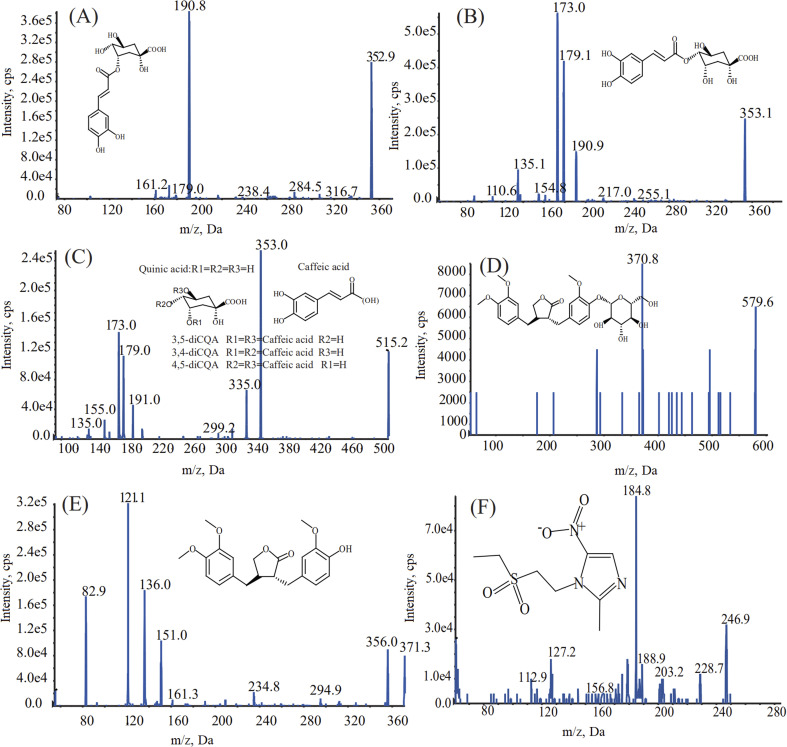
Chemical structure and MS/MS spectrograms of chlorogenic acid (A), cryptochlorogenic acid (B), isochlorogenic acid A, B, C (C), arctiin (D), arctigenin (E), and the internal standard, tinidazole (F).

The chromatographic conditions, including types of reversed phase chromatographic column, mobile phase compositions, choice of additives, column temperature, and flow rate of mobile phase, were optimized to achieve a short retention time, symmetric peak shape, and satisfactory ionization. In terms of the MS conditions, the responses of the analytes were superior in multiple-reaction monitoring (MRM) with negative ion mode. The optimized MS conditions are shown in Fig. 1 and Table 1. When acetonitrile and 0.1% formic acid–water were selected with a flow rate of 0.3 mL mL^−1^, the seven compounds showed better separation and a higher response.

**Table 1:** The MS/MS detection parameters, and the regression data of the seven compounds

Compounds	Retention time (min)	MRM transitions	DP (V)	Collision energy (V)	Regression equations	*r* ^2^
Chlorogenic acid	3.11	352.9 → 190.8	−34.76	−24.61	*y* = 0.0178*x* + 0.1286	0.9985
Cryptochlorogenic acid	3.20	353.1 → 173.0	−80.19	−25.05	*y* = 0.0156*x* − 0.0408	0.9993
Isochlorogenic acid B	4.48	515.2 → 353.0	−47.79	−20.12	*y* = 0.0188*x* − 0.0139	0.9996
Isochlorogenic acid A	4.64	515.2 → 353.0	−47.79	−20.12	*y* = 0.0170*x* + 0.0331	0.9990
Isochlorogenic acid C	4.88	515.2 → 353.0	−47.79	−20.12	*y* = 0.0101*x* − 0.0031	0.9997
Arctiin	5.51	579.6 → 370.8	−80.01	−24.68	*y* = 0.0004*x* + 0.0231	0.9991
Arctigenin	6.33	371.3 → 136.0	−78.99	−33.36	*y* = 0.0008*x* + 0.0162	0.9994

### 3.3. Method validations

#### 3.3.1. Linearity and LLOQ

The values of the linearity range included 2 to 1250 ng mL^−1^ for 3-CQA, 2 to 2000 ng mL^−1^ for 4-CQA, 1 to 2000 ng mL^−1^ for 3,5-diCQA, 1 to 500 ng mL^−1^ for 3,4-diCQA, 1 to 500 ng mL^−1^ for 4,5-diCQA, 2 to 10 000 ng mL^−1^ for ARC, and 2 to 2000 ng mL^−1^ for ARG. In particular, each of the correlation coefficients obtained was greater than 0.998. Furthermore, the LLOQ of 3-CQA, 4-CQA, 3,5-diCQA, 3,4-diCQA, 4,5-diCQA, ARC, and ARG were 2, 2, 1, 1, 1, 2 and 2 ng mL^−1^, respectively.

#### 3.3.2. Selectivity

**Fig. 2:**
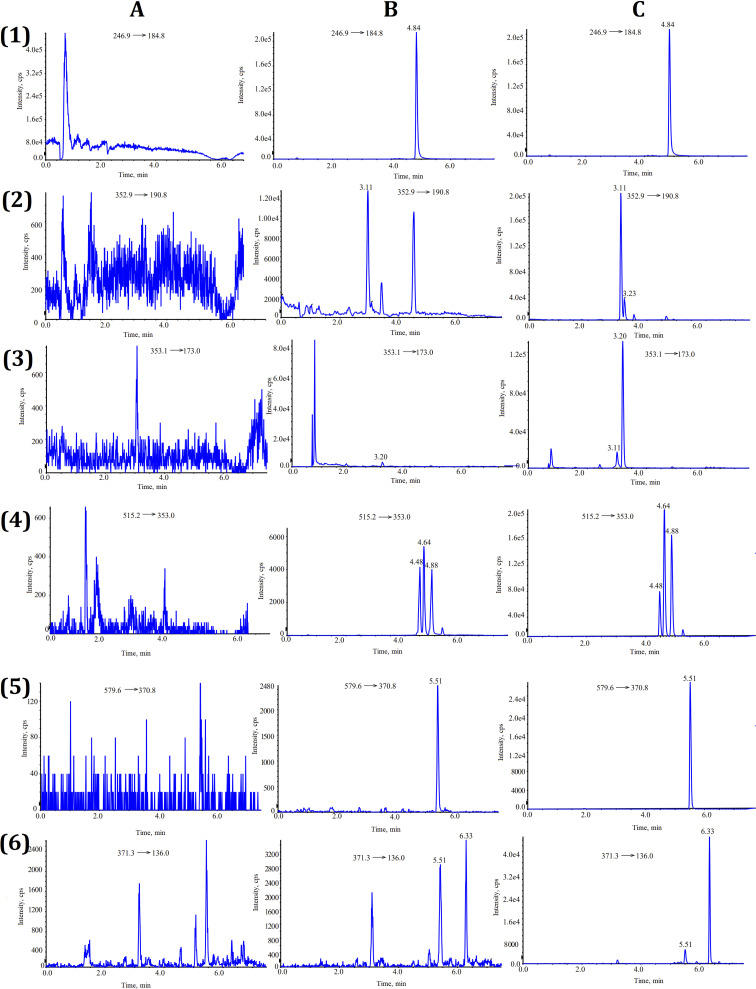
Representative chromatograms of (A) blank plasma, (B) blank plasma spiked with standard compounds, and (C) plasma samples. (1) Tinidazole, (2) chlorogenic acid, (3) cryptochlorogenic acid, (4) isochlorogenic acid A, B, C, (5) arctiin, and (6) arctigenin.

The selectivity was validated by determining the blank plasma from seven different lots and comparing the MRM chromatographic profiles of the plasma samples, which were spiked with the seven compounds. As shown in Fig. 2, excellent separation and lack of interference from endogenous components for all analytes is apparent.

#### 3.3.3. Accuracy and precision

As shown in Table 2, the precision level fell below 10.0% and the recovery range of the QC samples was 61.1% to 90.0% across three diverse levels for intra-day and inter-day values. Consequently, the degree of reproducibility and coherence of the proposed method was confirmed. The RSD results were within the FDA bioanalytical method validation guidance limits (±15% for QC samples and ±20% for LLOQ), which demonstrated satisfactory precision, accuracy, and reproducibility.

**Table 2:** Precision, accuracy, and recovery of the seven compounds (*n* = 6)

Compounds	Concentration (ng mL^−1^)	Precision (%)		Accuracy (%)		Recovery (%)	
		Inter-day	Intra-day	Inter-day	Intra-day	Accuracy	RSD
Chlorogenic acid	5	4.31	3.41	6.60	8.80	77.81	10.98
	250	2.94	3.15	4.45	5.80	64.29	6.09
	1000	3.57	1.77	3.41	3.12	69.73	3.23
Cryptochlorogenic acid	5	3.33	4.83	8.60	8.20	86.05	5.53
	400	2.39	2.69	5.29	6.81	61.07	5.31
	1600	5.23	5.37	5.49	6.03	68.36	9.66
Isochlorogenic acid B	2	6.16	5.77	6.50	5.50	72.13	4.31
	100	6.79	9.48	4.57	4.33	76.02	1.25
	400	4.38	3.96	3.84	3.58	77.93	5.25
Isochlorogenic acid A	2	6.34	4.29	7.50	8.00	63.75	4.88
	400	4.50	2.21	5.78	7.77	71.69	4.67
	1600	6.13	6.70	6.32	6.15	75.01	7.61
Isochlorogenic acid C	2	6.37	3.45	7.00	6.50	83.47	5.37
	100	5.16	3.37	7.19	5.22	77.77	4.29
	400	1.85	2.60	6.42	5.53	75.96	6.21
Arctiin	5	4.29	6.72	5.80	5.00	89.38	4.99
	2000	8.49	9.67	5.27	4.38	82.30	3.16
	8000	7.94	8.16	4.43	4.03	90.05	5.18
Arctigenin	5	4.11	2.95	8.20	7.60	76.07	7.74
	400	6.89	4.27	8.03	6.29	71.37	8.36
	1600	6.68	9.19	6.45	6.20	68.11	6.28

#### 3.3.4. Stability

As shown in Table 3, the range of accuracy is 90.0% to 110.0%, whereas the RSD of the seven compounds proved to be stable under the designated conditions of three freeze–thaw cycles, *i.e.*, retention in an auto-sampler (4 °C) for 4 h or 24 h, and storage at −80 °C for 1 month.

**Table 3:** Stability of the seven compounds (*n* = 6)

Compounds	Concentration (ng mL^−1^)	Freeze–Thaw cycles		Auto-sampler for 4 h		Auto-sampler for 24 h		−80 °C for 1 month	
		Mean ± SD	RE (%)	Mean ± SD	RE (%)	Mean ± SD	RE (%)	Mean ± SD	RE (%)
Chlorogenic acid	5	5.34 ± 0.23	6.80	5.28 ± 0.18	5.60	5.35 ± 0.21	7.00	5.31 ± 0.31	6.20
	250	265.31 ± 7.81	6.12	258.92 ± 8.16	3.57	261.20 ± 10.01	4.48	261.45 ± 9.94	4.58
	1000	1042.21 ± 37.23	4.22	1036.11 ± 18.65	3.61	1025.31 ± 37.11	2.53	1031.52 ± 29.01	3.15
Cryptochlorogenic acid	5	5.41 ± 0.17	8.20	5.36 ± 0.25	7.20	5.39 ± 0.12	7.80	5.31 ± 0.28	6.20
	400	427.02 ± 10.19	6.76	418.23 ± 11.23	4.56	420.79 ± 12.31	5.20	421.03 ± 14.31	5.26
	1600	1508.25 ± 78.93	5.73	1553.21 ± 83.45	2.92	1553.13 ± 65.02	2.93	1561.24 ± 54.34	2.42
Isochlorogenic acid B	2	2.11 ± 0.13	5.50	2.14 ± 0.12	7.00	2.16 ± 0.16	8.00	2.15 ± 0.12	7.50
	100	94.95 ± 6.67	5.05	96.54 ± 9.15	3.46	95.41 ± 8.81	4.59	96.13 ± 7.22	3.87
	400	419.01 ± 18.34	4.75	414.44 ± 16.55	3.61	413.55 ± 17.09	3.39	415.67 ± 23.41	3.92
Isochlorogenic acid A	2	2.08 ± 0.13	4.00	2.10 ± 0.09	5.00	2.11 ± 0.12	5.50	2.17 ± 0.12	8.50
	400	411.12 ± 18.52	2.78	412.03 ± 9.11	3.01	414.65 ± 18.13	3.66	416.98 ± 21.13	4.25
	1600	1651.25 ± 101.23	3.20	1658.93 ± 111.15	3.68	1671.21 ± 120.93	4.45	1654.33 ± 78.91	3.40
Isochlorogenic acid C	2	2.14 ± 0.13	7.00	2.18 ± 0.07	9.00	2.17 ± 0.17	8.50	2.14 ± 0.13	7.00
	100	105.61 ± 5.45	5.61	104.78 ± 3.53	4.78	104.12 ± 8.34	4.12	104.55 ± 6.07	4.55
	400	422.13 ± 7.81	5.53	420.01 ± 10.93	5.00	421.62 ± 11.34	5.41	420.98 ± 13.21	5.25
Arctiin	5	4.69 ± 0.21	6.20	4.61 ± 0.33	7.80	4.88 ± 0.27	2.40	4.63 ± 0.18	7.40
	2000	2080.86 ± 176.60	4.04	2081.22 ± 201.23	4.06	2100.56 ± 188.91	5.03	2084.23 ± 145.30	4.21
	8000	8241.91 ± 654.20	3.02	8300.19 ± 677.32	3.75	8311.71 ± 453.98	3.90	8281.27 ± 461.10	3.52
Arctigenin	5	5.38 ± 0.21	7.60	5.29 ± 0.15	5.80	5.30 ± 0.31	6.00	5.35 ± 0.08	7.00
	400	413.51 ± 28.51	3.38	415.01 ± 17.71	3.75	420.01 ± 10.52	5.00	415.22 ± 33.05	3.81
	1600	1648.43 ± 112.79	5.53	1650.12 ± 154.32	3.13	1701.23 ± 99.62	6.33	1687.91 ± 87.91	5.49

#### 3.3.5. Matrix effects and recoveries

As shown in Table 2, the recoveries of the seven compounds ranged from 61.07% to 90.05%, whereas the RSD was below 10.98% at three concentration levels. In addition, the matrix effects of the six compounds fell within the range of 85.0% to 117.0%, whereas the RSD was below the threshold of 13.0% at three concentrations.

### 3.4. Pharmacokinetic application

**Fig. 3:**
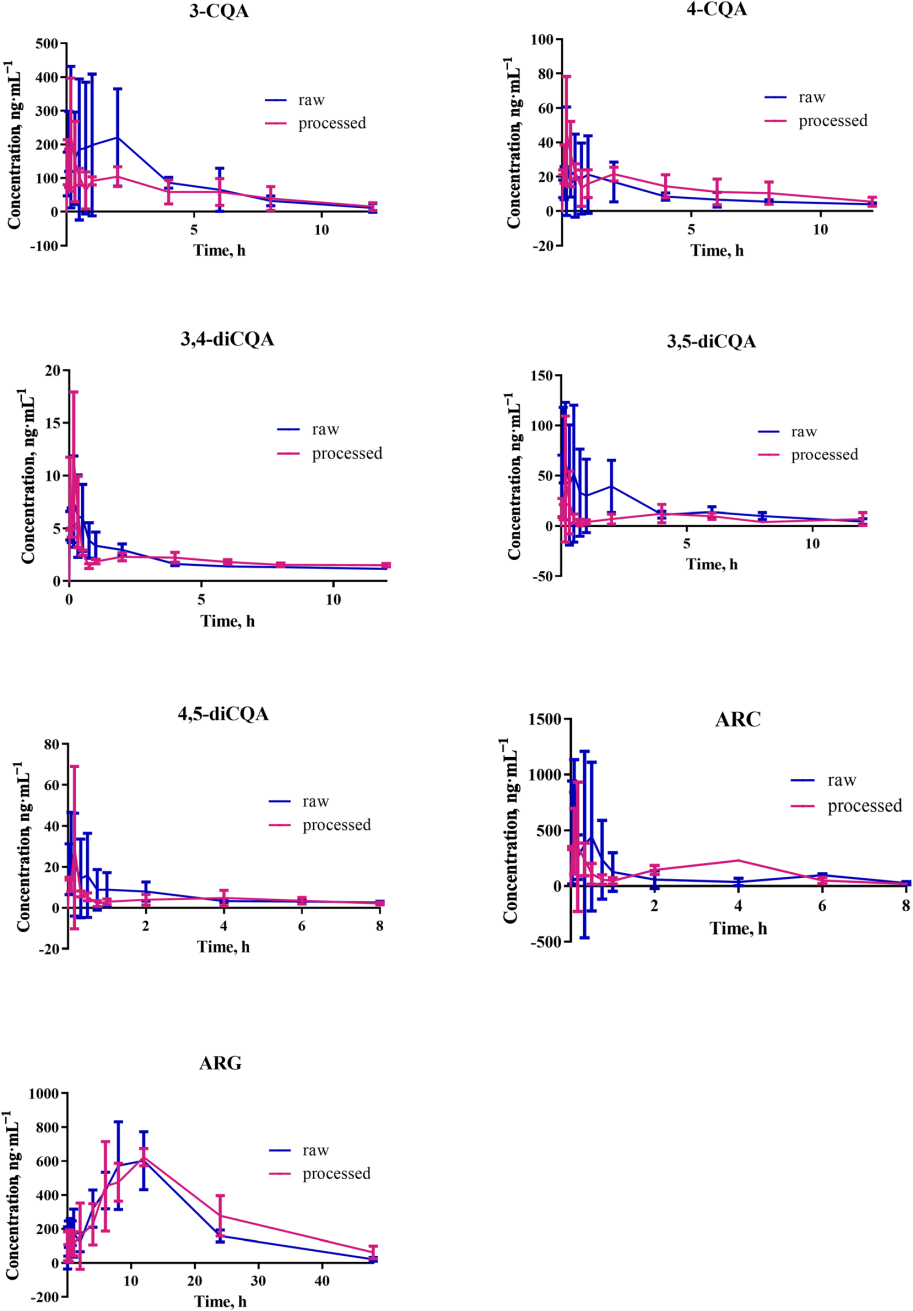
The mean plasma concentration time profiles of seven compounds after oral administration of RAF and SAF (*n* = 6).

The proposed method was employed to scrutinize the pharmacokinetics of the seven compounds to ensure safe oral administration of the RAF and SAF extracts. The pharmacokinetic profiles of the seven compounds were portrayed in a one-compartment model, with the mean plasma concentration time-curve outliers being shown in Fig. 3. As is evident from the results given in Table 4, the area under the curve for 0 to 24 h (AUC_(0–24h)_) of ARG was greater in all the compounds of the SAF, which indicated the frequency of abundant plasma exposure. In addition, the AUC_(0–24h)_ and *C*_max_ of 3,4-diCQA and 4,5-diCQA were lower than those of other compounds in the RAF and SAF, which suggests there were weaker absorption levels *in vivo*. Moreover, the ARG exhibited the double-peak phenomenon, which was inextricably linked to the transformation between the ARC and ARG. Using in-depth comparisons of the pharmacokinetic parameters of RAF and SAF (Table 4), the *T*_max_ values of the seven compounds after oral administration of RAF extract modestly extended than that of the SAF extract. Overall, these results indicated that there was a higher degree of absorption of the seven compounds that underwent the stir-frying process. Furthermore, the *C*_max_, AUC_(0–24h)_, and the area under the curve from 0 extrapolated to infinity (AUC_(0–∞)_) of 4-CQA, ARC and 3,4-diCQA exhibited remarkable differences between RAF and SAF, thereby showcasing the dynamic nature of the absorption in the treatment process.^16^ Similarly, the AUC_(0–24h)_ of ARC were 1074.25 ± 416.8 and 1967.51 ± 599.5 ng mL^−1^ for the RAF and SAF samples, respectively. From the study, a discernible increase in exposure of plasma to ARC from the AF was apparent following processing. Furthermore, the current study underlines the potential growth in bioavailability of ARC and the accelerated rate of the primary seven compound adsorption levels through the stages of stir-frying.^17,18^ In particular, the likely alteration of composition proportions in relation to the herbal extract and pharmacokinetic interactions of multiple components *in vivo* might be the key factor. However, whether this assertion is correct remains to be confirmed by further research.

**Table 4:** Pharmacokinetic parameters of seven compounds after oral administration of RAF and SAF (*n* = 6)

Compounds	Samples	*t*_1/2_ (h)	AUC_(0–*t*)_ (μg L^−1^ h^−1^)	AUC_(0–∞)_ (μg L^−1^ h^−1^)	*C*_(max)_ (μg L^−1^)	*T*_max_ (h)	Mean residence time (MRT_(0–∞)_) (h)	Clearance (CL) (L h^−1^ kg^−1^)
Chlorogenic acid	RAF	2.175 ± 0.28	1071.09 ± 594.4	1087.3 ± 588.2	348.5 ± 161.3	0.875 ± 0.41	3.719 ± 0.60	13 532.2 ± 6150.8
	SAF	4.371 ± 0.32	694.97 ± 307.1	936.9 ± 659.7	303.7 ± 105.7	0.111 ± 0.43	5.090 ± 1.82	17 674.6 ± 8956.3
Cryptochlorogenic acid	RAF	12.61 ± 7.96	152.32 ± 60.74	196.19 ± 59.14	34.80 ± 25.06	0.431 ± 0.07	19.86 ± 9.37	65 738.2 ± 18 603.4
	SAF	13.49 ± 7.98	218.15 ± 80.28	279.99 ± 68.02	116.16 ± 35.41	0.103 ± 0.05	16.93 ± 8.60	45 049.3 ± 10 999.7
Isochlorogenic acid B	RAF	35.68 ± 18.24	38.17 ± 13.89	97.95 ± 46.47	11.33 ± 8.68	0.117 ± 0.07	52.97 ± 27.74	139 632.1 ± 45 862.4
	SAF	24.27 ± 11.54	43.77 ± 13.25	81.42 ± 25.31	43.22 ± 26.80	0.103 ± 0.05	32.75 ± 15.17	161 334.3 ± 55 171.3
Isochlorogenic acid A	RAF	6.704 ± 3.61	218.56 ± 109.9	271.7 ± 168.8	99.05 ± 37.81	0.792 ± 0.09	8.352 ± 2.91	59 654.7 ± 5655.3
	SAF	3.265 ± 1.26	101.31 ± 73.10	180.2 ± 63.60	135.71 ± 25.50	0.100 ± 0.07	7.967 ± 5.12	182 852.5 ± 28 334.8
Isochlorogenic acid C	RAF	9.002 ± 5.89	71.53 ± 33.22	84.11 ± 46.26	39.89 ± 17.30	0.125 ± 0.05	15.89 ± 11.25	168 351.6 ± 60 078.5
	SAF	6.770 ± 4.30	79.51 ± 25.10	198.8 ± 93.93	103.84 ± 47.62	0.078 ± 0.03	8.736 ± 5.55	103 760.6 ± 66 088.5
Arctiin	RAF	2.995 ± 1.42	826.5 ± 216.4	1074.2 ± 416.8	1019.3 ± 605.9	0.152 ± 0.09	5.328 ± 2.99	20 510.8 ± 14 945.5
	SAF	3.834 ± 1.86	942.7 ± 228.2	1967.5 ± 599.5	605.7 ± 389.5	0.072 ± 0.05	8.982 ± 3.11	6531.4 ± 1755.3
Arctigenin	RAF	8.249 ± 1.09	11 567.8 ± 2257.8	11 833.5 ± 2335.2	706.9 ± 148.9	9.000 ± 2.45	14.89 ± 0.99	1055.5 ± 254.7
	SAF	9.953 ± 4.46	11 985.2 ± 5706.5	13 114.6 ± 6079.4	600.8 ± 158.3	8.667 ± 3.72	17.83 ± 6.08	1459.4 ± 507.6

## 4. Conclusions

To summarize, a sensitive and streamlined method was formulated with the aim of simultaneous quantification of compounds such as 3-CQA, 4-CQA, 3,5-diCQA, 3,4-diCQA, 4,5-diCQA, ARC, and ARG following the stages of oral administration of RAF and SAF extracts in rats. This method was successfully used in a pharmacokinetic study. This method will also be valuable for use in human clinical studies in the future, because it should be possible to obtain even higher sensitivity than that previously reported. Surprisingly, the pharmacokinetic data demonstrated that the stir-frying process expedited the degree of adsorption of the seven compounds and enriched the bioavailability of ARC. Finally, the scope of research was primarily intended for the assessment of pharmacokinetics of multiple, orally administrated compounds of RAF and SAF extracts to rats. Nevertheless, the key findings of this study will be beneficial for expanding their practical applications and effectively understanding the stir-frying mechanism.

## Ethical statement

All animal procedures were performed in accordance with the Guidelines for Care and Use of Laboratory Animals of Nanjing University of Chinese Medicine, and approved by the Animal Ethics Committee of Nanjing University of Chinese Medicine.

## Conflicts of interest

The authors declare that they have no known competing financial interests or personal relationships that could have appeared to influence the work reported in this paper.

## Supplementary Material
